# Eggshell membrane and green seaweed (Ulva *lactuca*) micronized powders for in vivo diabetic wound healing in albino rats: a comparative study

**DOI:** 10.1186/s40780-024-00345-x

**Published:** 2024-07-23

**Authors:** Moustafa H. Moustafa, Mohamed S. Turkey, Noha S. Mohamedin, Amira A. Darwish, Amira A. M. Elshal, Mona A. H. Yehia, Mohamed M. El Safwany, Ehab I. Mohamed

**Affiliations:** 1 Medical Biophysics Department, Medical Research Institute, Alexandria University, 165 El-Horreya Avenue, Alexandria, 21561 Egypt; 2 Microbiology and Immunology Department, Faculty of Pharmacy, October 6 University, Sixth of October City, Giza, Egypt; 3 Medical Laboratory Technology Department, Faculty of Applied Health Sciences Technology, Pharos University, Alexandria, Egypt; 4 Histochemistry and Cell Biology Department, Medical Research Institute, Alexandria University, Alexandria, Egypt; 5 Radiological Sciences and Medical Imaging Department, Faculty of Applied Health Sciences Technology, Pharos University, Alexandria, Egypt

**Keywords:** Eggshell membrane (ESM), Seaweed (Ulva *lactuca*), Induced-diabetes wound, Wound healing, Compressive strength, Dielectric constant

## Abstract

**Background:**

Nonhealing diabetic wounds are a serious complication associated with extremely lethargic wound closure and a high risk of infection, leading to amputation or limb loss, as well as substantial health care costs and a poor quality of life for the patient. The effects of either eggshell membrane (ESM) and green seaweed (Ulva *lactuca*) extracts alone or in combination were evaluated for in vivo skin wound healing in a rat model of induced diabetes.

**Methods:**

Micronized powders of waste hen ESM, Ulva *lactuca*, and their 1:1 mixture were prepared using regular procedures. The mechanical, electrical, and surface morphology characteristics of powders were examined using direct compression, LCR-impedancemetry, and scanning electron microscopy. The effect of ESM, Ulva *lactuca*, and their mixture as compared to standard Dermazin treatments were evaluated on wounds inflicted on male Wistar Albino rats with induced diabetes. Quantitative wound healing rates at baseline and at 3, 7, 14, and 21 days of treatments among all rat groups were conducted using ANOVA. Qualitative histological analysis of epidermal re-epithelization, keratinocytes, basement membrane, infiltrating lymphocytes, collagen fibrines, and blood vessels at day 21 were performed using Image J processing program.

**Results:**

Compressive strength measurements of tablets showed a Young’s modulus of 44.14 and 27.17 MPa for the ESM and ESM + Ulva *lactuca* mixture, respectively. Moreover, both samples exhibited relatively low relative permittivity values of 6.62 and 6.95 at 1 MHz, respectively, due to the porous surface morphology of ESM shown by scanning electron microscopy. On day 21, rats treated with ESM had a complete diabetic wound closure, hair regrowth, and a healing rate of 99.49%, compared to 96.79% for Dermazin, 87.05% for Ulva *lactuca*, 90.23% for the mixture, and only 36.44% for the negative controls. A well-formed basement membrane, well-differentiated epithelial cells, and regular thick keratinocytes lining the surface of the epidermal cells accompanied wound healing in rats treated with ESM, which was significantly better than in control rats.

**Conclusion:**

Ground hen ESM powder, a low-cost effective biomaterial, is better than Ulva *lactuca* or their mixture for preventing tissue damage and promoting diabetic wound healing, in addition to various biomedical applications.

## Introduction

Diabetes is a worldwide epidemic, particularly in developing North African countries like Egypt, which ranks 9th in the world with a prevalence of 15.56% among adults [1, 2]. The International Diabetes Federation reports that 73 million adults in the Middle East and North Africa region, including 796,000 deaths in 2021, are currently living with diabetes, predicted to rise to 95 million by 2030 and to reach 136 million by 2045 [2]. Over the past 15 years, global healthcare costs related to diabetes have increased by 316%, to a total of at least $966 billion [2]. Chronic nonhealing wounds (e.g., diabetic, venous, and pressure ulcers) are a prevalent and costly health burden that can result in substantial morbidity and mortality [3, 4]. Wound healing is stalled by a plethora of variables, including diabetes-related neuropathy, ischemia, infection, hyperglycemia-induced inflammation, malnutrition, and oxidative stress [5]. It is critical, then, to develop more effective therapeutic modalities for diabetic wound healing to prevent serious complications such as infection and amputation [4].

Customized approaches to wound tissue regeneration are possible thanks to the use of new technologies and novel biomaterials with distinct features [4, 6]. Electrical stimulation, ultrasound, and compression therapy are examples of physical modalities used as adjuvants to promote wound healing with fewer complications [7–9]. Emerging new interventions like autografting, stem cell replacement, nano-biomaterials, and natural product remedies, together with analgesics and anti-toxins, are also being considered to treat dermatologic conditions, like cuts, wounds, and infections [6, 10, 11]. Inert biomaterials have evolved to incorporate biological components like nanostructure and cytokines/growth factors to mimic tissue properties [4, 6]. Scientists are working on programmable matrices that can integrate endogenous inputs and have biomechanical, bioelectrical, and drug delivery characteristics that are customized to certain tissues or biomedical applications [6].

In this context, eggshell membrane (ESM), a natural biomaterial with calcium, collagens, hyaluronic acid, and chondroitin sulfates, has potential as a natural bandage for treating cutaneous wounds and burns due to its exceptional physical and mechanical properties [12–16]. ESM showed promising results as a wound treatment due to its analgesic effects, network structure, permeability, moisture retention, and ability to promote wound re-epithelialization [14–16]. That is, tissue regeneration of superficial open wounds in male Albino rats was significantly enhanced by the application of ESM powder and dressing in comparison with the conventional treatment using hydrogen peroxide [14]. Moreover, ESM has been studied in vitro for its potential as a scaffold for wound healing and pain mitigation at areas of skin grafts, a tissue displacement material, bone regeneration, prosthesis, implants, plastic surgery, and corneal reconstruction procedures [6, 9, 11, 15, 16]. Furthermore, ESM microfibers coated with silver nanoparticles promoted re-epithelialization, granulation tissue formation, and wound healing by regulating inflammation response and increasing cell proliferation in an Albino mouse experimental model [12, 17]. Recent in vitro research with human dermal fibroblast cell lines showed that ESM combined with silver nanoparticles exhibited promising antibacterial, biological, mechanical, and physical properties for dermal wound healing [18].

Seaweed extracts, rich in bioactive compounds like polysaccharides, oligosaccharides, polyunsaturated fatty acids, sterols, proteins, polyphenols, carotenoids, vitamins, and minerals, may protect against tumors, infections, inflammation, diabetes, and immune system abnormalities [19–21]. Evidence suggests that Sargassum *ilicifolium* and Ulva *lactuca* can improve wound healing and decrease tissue damage in both in vitro and in vivo studies [19]. That is, L929 cells treated with Sargassum *ilicifolium* extracts showed higher cell proliferation and migration than those treated with Ulva *lactuca* extracts in vitro. In addition, Albino mice that were given aqueous Sargassum *ilicifolium* or Ulva *lactuca* extracts orally, rather than topically, showed markedly improved wound healing. Furthermore, brown algae’s Sargassum *fusiforme* fucoidan and green algae’s Ulva *lactuca* hydroethanolic extracts showed anti-diabetic effects, suppressing inflammation, hyperglycemia, hypercholesterolemia, and hyperlipidemia levels, and enhancing insulin sensitivity in streptozotocin and alloxan-induced diabetes in Albino rats [20, 22, 23]. Based on these findings, we propose that ESM and Ulva *lactuca* be evaluated in an experimental rat model of diabetic wound healing. Research comparing the efficacy of topical skin ESM and Ulva *lactuca* treatments for diabetic wound healing in rats is, however, severely insufficient.

The present comparative study aims to assess the effects of individual and mixture ESM and green seaweed (Ulva *lactuca*) sterile micronized powders on in vivo skin wound healing in a rat model of induced diabetes.

## Materials and methods

### Eggshell membrane powder preparation

Waste household hen ESMs were recovered and washed twice with regular and deionized water to eliminate any remaining egg white residue [24]. The ESMs were boiled for 30 min followed by a hot air oven drying process at 80 °C for 2 h. The ESMs were first crushed using a mortar and pestle and then ground into a micronized powder (< 20 μm) in a grinder to increase its bioavailability [25]. Finally, the ESM powder was UV-sterilized and stored for later use.

### Ulva *lactuca* powder preparation

The fresh green seaweeds Ulva *lactuca* were harvested from the Mediterranean Sea coast, Alexandria, Egypt, during spring season 2022 [23, 26]. The fresh Ulva *lactuca* was soaked in three times its weight in tap water to eliminate any lingering salt and residues, then rinsed with running water and finally with deionized water. After being air dried in the shade for 5–8 days, or until they are readily broken by hand, the Ulva *lactuca* was ground into a fine powder using an electric blender and then a mortar and pestle. The Ulva *lactuca* powder was finally UV-sterilized and stored.

### Mechanical measurements (compressive strength)

Tablets of ESM powder and ESM + Ulva *lactuca* mixture (1:1, *w*/*w*) were prepared by direct compression using a circular 15 mm diameter flat-faced punches on a rotating tablet compressing machine (Model: R190FT; GEA-Courtoy, Halle, Belgium) to investigate their mechanical characteristics [27]. The ESM and ESM + Ulva *lactuca* mixture tablets compressive strength was measured by their resistance to size reduction due to applied simultaneous axial and radial loads. To determine the viscoelastic characteristics, tablets of each sample were placed consecutively in the bottom ring of the compressive test machine (Model: H10KS, Tinius Olsen, USA), and a force of 1676.85 N and 2087.91 N, respectively, was applied to monitor graphically their stress-strain behavior.

### Electrical measurements (dielectric properties)

The dielectric constant (i.e., relative permittivity) is a mathematical measure of a substance’s ability to store electrical energy and concentrate electric flux, calculated by comparing its permittivity to that of vacuum. An LCR-impedance meter (Fluke, *PM6306* + cable *PM9541b*, Test Equipment Solutions Ltd., UK) was used to measure the dielectric properties of the samples at room temperature of 25 ± 2 °C. ESM and ESM + Ulva *lactuca* mixture powder samples were placed between the silver electrodes on the printed circuit board of the LCR test cell and measurements were taken. Samples’ relative permittivity (*έ*) was calculated by measuring their capacitance (*C*) in the frequency range of 1 kHz – 1 MHz [28].

The real part of each sample relative permittivity was calculated using the relation:

1 $$\acute{\varepsilon}=\mathrm{C} / \mathrm{C}_o$$

Where *C*_*o*_ is the test cell capacitance in the air, which is given by relation:

2 $$\mathrm{C}_o=\varepsilon_o \mathrm{ \ A} / \mathrm{d}$$

Where *ε*_*o*_ is the vacuum (∼ air) permittivity, *A* is the cell wall area, and *d* is the distance between the cell electrodes [29].

### Scanning electron microscopy (SEM)

The powders of ESM and ESM + Ulva *lactuca* mixture were coated with a sputter coating evaporator (Model: SPI Module, Sputter Carbon/Gold Coater). The morphology of their surfaces was examined at magnifications of 1,000*×* and 2,500*×* using a scanning electron microscope (SEM) (JSM-IT200 Series, InTouchScope™, JEOL, Japan) running at 30 kV acceleration voltage.

### Diabetic wound healing in vivo examination

#### Experimental design

Adult diabetic male Wistar Albino rats (*n* = 25) had 15–23 mm long incisions made in the panniculus carnosus layer of their epidermis and were randomly divided into five equal groups (*n* = 5) for the purpose of the experiment. Over a 21-day period, wounded rats underwent sham and four different skin topical treatments, as follows: Group I consisted of untreated wound negative controls, Group II of positive control animals treated with 1% Silver Sulfadiazine cream (Dermazin), Group III of animals treated with ESM powder, Group IV of animals treated with Ulva *lactuca* powder, and Group V of animals treated with ESM + Ulva *lactuca* mixture.

#### Animals

A total of 25 healthy male Wistar Albino rats, which were seven months old and weighed between 250 and 350 g when we started the experiment, were randomly divided into five equal groups. Each group of rats was given a week to acclimatize to the natural daylight cycle in big quadrilateral cages of length 49, width 34, and height 16 cm, which could hold up to 5 adult rats. The rats were housed in an environment with a controlled temperature of 22 ± 2 °C and provided with water and regular rat chow [30]. The Institutional Animal Care and Use Committee and the Ethics Committee of the Medical Research Institute, Alexandria University, Alexandria, Egypt evaluated and approved the study (IACUC #0121950812) to ensure its conformity with the guidelines set out by US National Institutes of Health’s Guide for the Care and Use of Laboratory Animals [30]. All the methods described here adhere to the standards set out by the ARRIVE guidelines.

#### Induction of diabetes

Prior to diabetes induction, the animals fasted for 18 h but had free access to water. A 150 mg/kg dose of newly prepared Alloxan Monohydrate in 0.9% normal saline was administered *i.p.* instantly to cause diabetes in male Wistar Albino rats [31]. For the following 24 h, animals were given 5% glucose in drinking water to counteract the Alloxan-induced lethal hypoglycemia. After 72 h, fasting blood glucose (FBG) levels were measured, which might reach 200 mg/dl. Hyperglycemia and glycosuria, with FBG levels between 200 and 260 mg/dl, were seen in the rats after two weeks [32].

#### Infliction of wounds

For this investigation, male diabetic Wistar Albino rats were anesthetized with 5% Isoflurane in 100% oxygen in an anesthetic chamber for 45 min [25, 30]. After that, they had their back hair shaved and skin incisions of 15–23 mm long and 1–2 mm deep were made in the panniculus carnosus layer of their epidermis, which is known to induce fast wound contraction upon damage [14, 25]. Incisions were photographed digitally, and wound area (in mm^2^) was instantly calculated by multiplying the longest length by the widest perpendicular width measurements using a digital vernier caliper (Vinca DCLA-0605, China) [33]. Rats were injected daily with a dosage of 0.01 mg/kg of Buprenorphine *s.c.* to induce light anesthesia and were treated with enough topical medication to thoroughly cover skin wounds completely, according to experimental design. The skin wound healing progress of all animal groups was documented with digital photographs and area measurements on baseline and on days 3, 7, 14, and 21 for all kinds of treatment.

#### Histological analysis

After 21 days of treatment, rats were euthanized by an anesthetic overdose of Pentobarbital at a dosage of 800 mg/kg, which is proven to cause unconsciousness and painless death, without distress or anxiety. Animal’s death was confirmed by cutting the jugular vein cause exsanguination [25, 30]. The cutaneous tissues of the entire wound and the surrounding normal tissues were taken and preserved in a 4% formaldehyde solution [34]. After being fixed in 10% formalin, the tissue samples were dehydrated using a graded alcohol series, embedded in paraffin, sliced longitudinally into serial Sects. 4-5-µm thick using a microtome, and last, stained with hematoxylin and eosin (H&E) [35–37]. To assess epidermal regeneration at wound sites in diabetic rats, images at a magnification of 200*×* were captured using an HD digital camera attached to a light microscope (Model BX41, Olympus, Japan). Images of six fields per slide for each rat were subjected to qualitative analysis of epidermal re-epithelization, keratinocytes, basement membrane, infiltrating lymphocytes, collagen fibrines, and blood vessels using Image J processing program (Ver. 1.54 g, NIH, USA) [35, 36].

#### Blinding and masking

Each animal was examined by seven researchers in the following way: two researchers (MHM, NSM) randomized the animals into groups and applied sham and wound treatment following the experimental design. Only these researchers knew which animal was given which treatment. One researcher (MMS) oversaw both anesthesia and euthanasia. Lastly, five researchers (MST, AAD, AAE, MAY, EIM), all of whom were blinded to the treatments, evaluated the outcomes of wound treatments and histology results.

#### Outcome measurements

All animals showed signs of improvement after 21 days of sham and designated wound treatment, including a reduction in wound area, epidermal regeneration at wound sites, and increased re-epithelialization rates and decreased epithelial gaps in histological analysis.

#### Statistical analysis

Qualitative and quantitative data analyses were done using the SPSS processing program (Version 16; Chicago, IL, USA). For normally distributed quantitative variables, the Analysis of Variance (ANOVA) test was used for multiple-group comparisons and the Post Hoc test (Tukey) was used for pairwise comparisons. The data were summarized using means and standard deviations (SDs), and statistical significance was shown by a *p* < 0.05.

## Results and discussion

New technologies and novel biomaterials can help address the prevalent and costly chronic diabetic wounds, enabling customized wound tissue regeneration approaches that can significantly reduce morbidity and mortality. The individual and combined effects of ESM and Ulva *lactuca* sterile extracts on wound healing in vivo in diabetic-induced rats were characterized and investigated using the following methodologies.

### Visual description

**Fig. 1:**
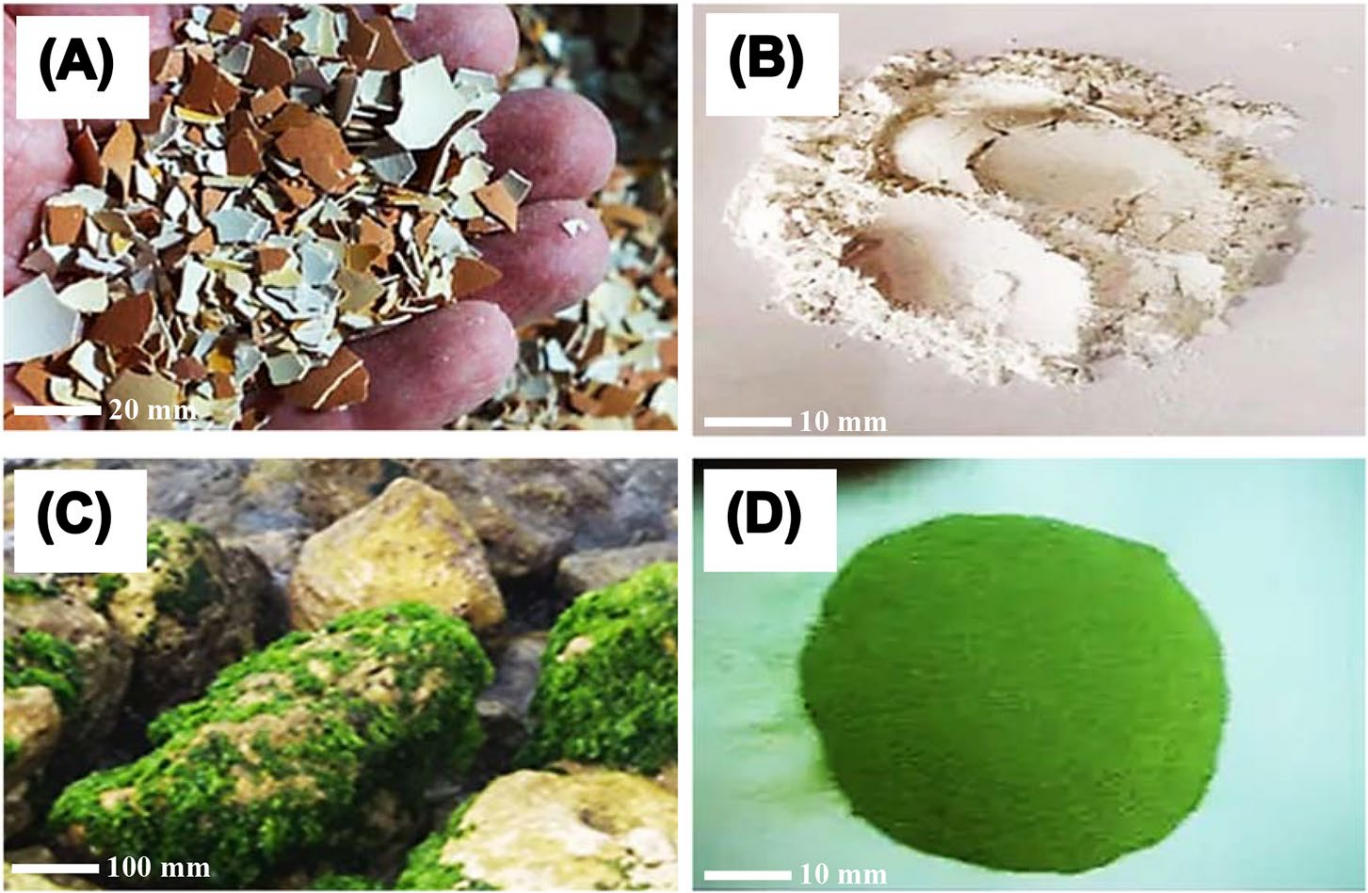
Visual appearance of the raw waste eggshell membrane (ESM) (**A** and **B**) and the green seaweed (Ulva *lactuca*) (**C** and **D**) before and after treatment and grinding

High purity product was obtained after extracting ESM and Ulva *lactuca* using standard procedures, as displayed in Fig. 1. Figure 1A and B show raw waste ESM both before and after treatment and grinding, revealing a typical white hue and a high degree of purity, respectively. Figure 1C and D show the final purity and characteristic green color of Ulva *lactuca*.

### Compressive strength

**Fig. 2:**
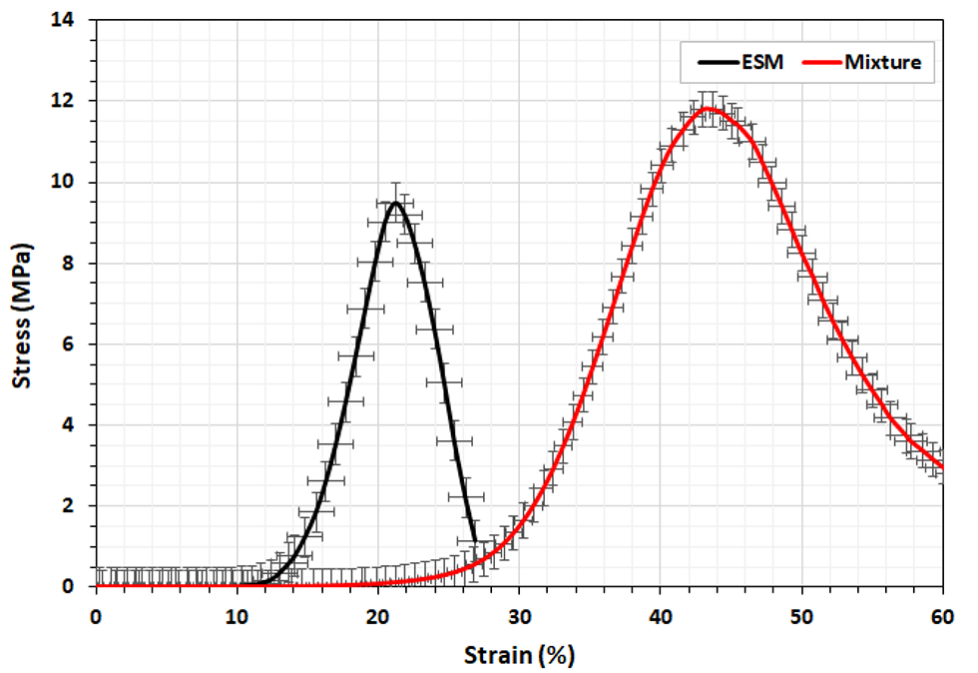
Stress-strain graphical behavior for the eggshell membrane (ESM) powder and the ESM + Ulva *lactuca* mixture samples showing a higher elastic modulus for the mixture at 11.82 MPa as compared to the ESM at 9.49 MPa

Compressive strength measurements were conducted on tablets of ESM and ESM + Ulva *lactuca* mixture since it was impossible to form tablets from Ulva *lactuca* powder due to its brittleness and stickiness. Table 1; Fig. 2 show the flexibility and elasticity ranges for the ESM and ESM + Ulva *lactuca* mixture. Figure 2 shows that, like other collagen-based systems, the stress-strain curves for both materials have a toe, a heel, and a linear dependence region, as recently described by Torres et al. [16]. Fiber un-folding and stretching at low stiffness due to entropic elasticity characterizes the toe region of collagen-based systems, while increased stiffness associated with membrane elongation characterizes the heel region, and a proportional elongation response to load is displayed in the linear dependence region [37, 38].

**Table 1:** Mean values of calculated mechanical properties for eggshell membrane (ESM) and the ESM + Ulva *lactuca* mixture tablets for compressive strength behavior

	ESM	Mixture
Diameter (mm)	15.00	15.00
Area (mm^2^)	177.00	177.00
Ultimate force (N)	1676.85	2087.91
Ultimate stress (MPa)	9.49	11.82
Break distance (mm)	1.35	3.07
Strain (%)	21.50	43.50
Young’s Modulus (MPa)	44.14	27.17

When the load levels are low, both materials exhibit a non-linear region in which the strain and applied stress are not directly related, and a linear region at higher levels in which strain and stress variations are directly related. After applying 1676.85 N, the ESM reached a maximum compressive strength of 9.49 MPa, while the ESM + Ulva *lactuca* mixture reached a maximum of 11.82 MPa after being subjected to 2087.91 N. Thus, the mixture is a biomaterial that is both more elastic and mechanically biocompatible, as evidenced by the average Young’s modulus values of 44.14 MPa for ESM and 27.17 MPa for the mixture [39]. Recent experimental measurements of hen ESM Young’s modulus have shown a wide range of variation between 4.2 and 38.1 MPa, with an average 19.8 ± 14.3 MPa, depending on the medium and the degree of dehydration of the ESM fibers [38]. It was argued that the glycoproteins-rich layer encapsulating a collagen core forms a basis for CaCO_3_ crystallization that is responsible for the observed differences in ESM Young’s modulus [40].

Most biopolymer materials, including the ESM and Ulva *lactuca*, exhibit viscoelastic mechanical characteristics that mimic the flexibility and resilience of their natural polymer and tissue counterparts. Collagen’s triple helix bundles provide animal tissues compressive and tensile strength and anchoring to cell adhesion via surface receptors [40]. Individual fibers’ activities and the type of their interactions, as in skin, cornea, tendon, and blood vessels, affect this behavior [16]. Putative mechano-responders in cells include membrane elements, cytoskeletal components, and the nucleus, and surrounding cell positioning in the tissue is determined by the mechanical microenvironment, which includes the extracellular matrix composition and extrinsic loading [41]. Moreover, the substrate stiffness has a direct impact on cell behavior, which in turn can modify the substrate itself and control the process of tissue regeneration [41, 42]. Thus, the inherent cell ability to detect external mechanical forces and substrate stiffness could benefit from biomaterials, such as ESM and ESM + Ulva *lactuca*, to direct stem or resident cells towards the process of tissue regeneration or repair [39, 42].

### Dielectric properties

**Fig. 3:**
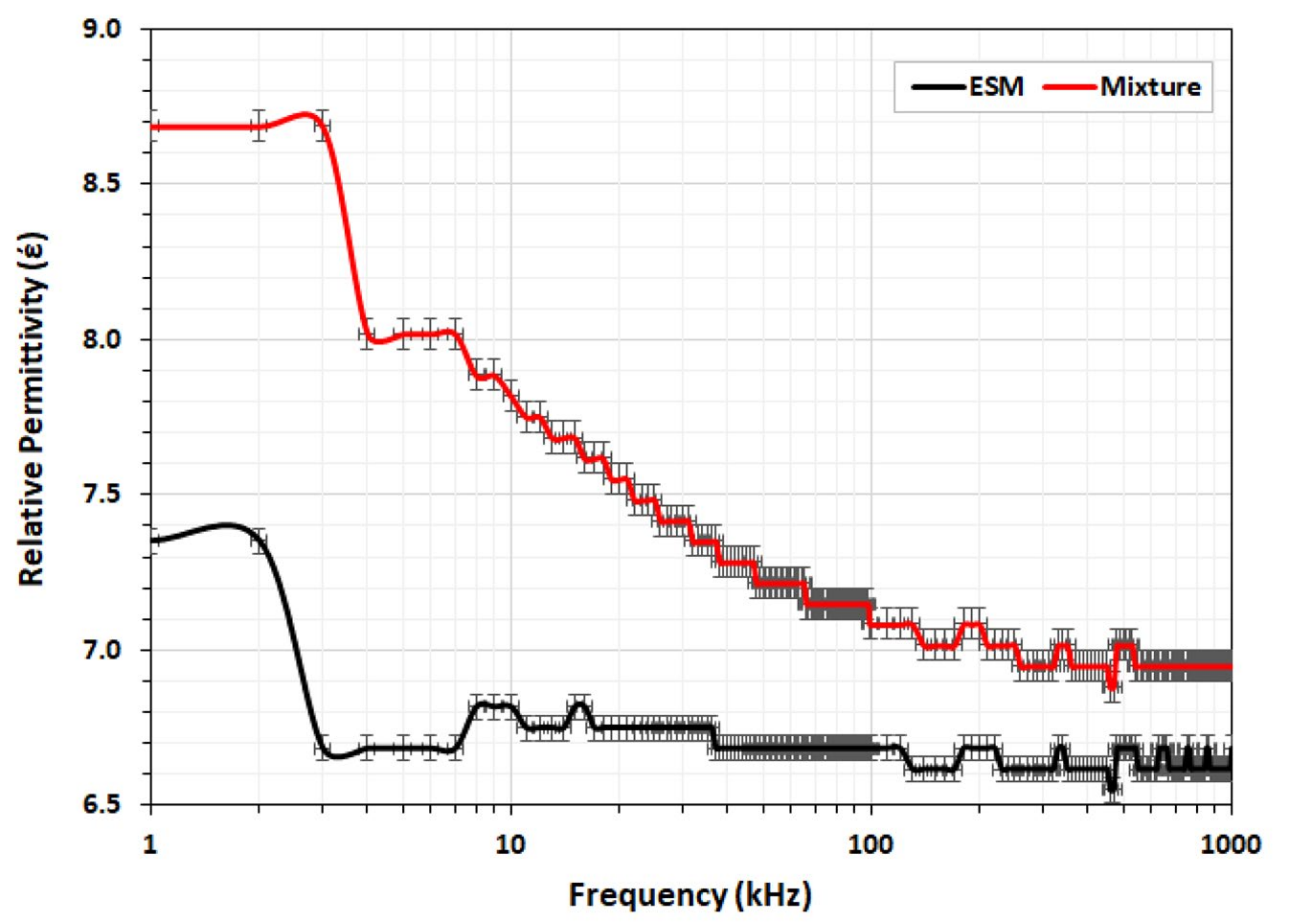
Variation of dielectric constant for the eggshell membrane (ESM) powder and the ESM + Ulva *lactuca* mixture samples in the frequency range from 1 kHz to 1 MHz

A material’s dielectric properties are frequency-dependent, among other factors like temperature and moisture content, since dipolar and ionic conduction mechanisms change as the frequency changes [24]. As shown in Fig. 3, the relative permittivity (έ) decreases from 7.35 to 6.62 for ESM and from 8.69 to 6.95 for ESM + Ulva *lactuca* mixture samples as the frequency increases from 1 kHz to 1 MHz at the same temperature (25 ± 2°C), due in part to the ESM’s low moisture content and to polarization effects [24, 43]. The overall relative permittivity values for both samples, plateauing at 6.62 and 6.95 at 1 MHz, respectively; are relatively small because of ESM porous nature [43], with a slightly higher value for the mixture due to Ulva *lactuca*. It’s worth noting that the ESM maintained a nearly constant relative permittivity of 6.62 throughout a wide frequency range (3 kHz – 1 MHz), making it an effective biomaterial for tissue engineering applications [6, 11–17, 25, 38, 40–43]. It was shown earlier that the hen ESM relative permittivity retained approximately constant values between 8.5 and 6.5 in the temperature range 25–35 °C, before dramatically reducing to 1.0 at 100 °C, at frequencies from 200 MHz to 20 GHz [25]. The relative permittivity tends to approach the value of a vacuum (i.e., 1.0) at very high frequencies because reorientation of the dipoles becomes impossible [44]. Lin et al., have recently measured a transferred charge density of 0.80 mC/m^2^, a surface potential of 4.5 kV, and a relative permittivity of 2.81 for hen ESM at 1 MHz, arguing that its rough surface morphology and non-flat 3D topography may decrease power production owing to inadequate draping over the friction layer in comparison to other avian species ESM [43].

### SEM analysis

**Fig. 4:**
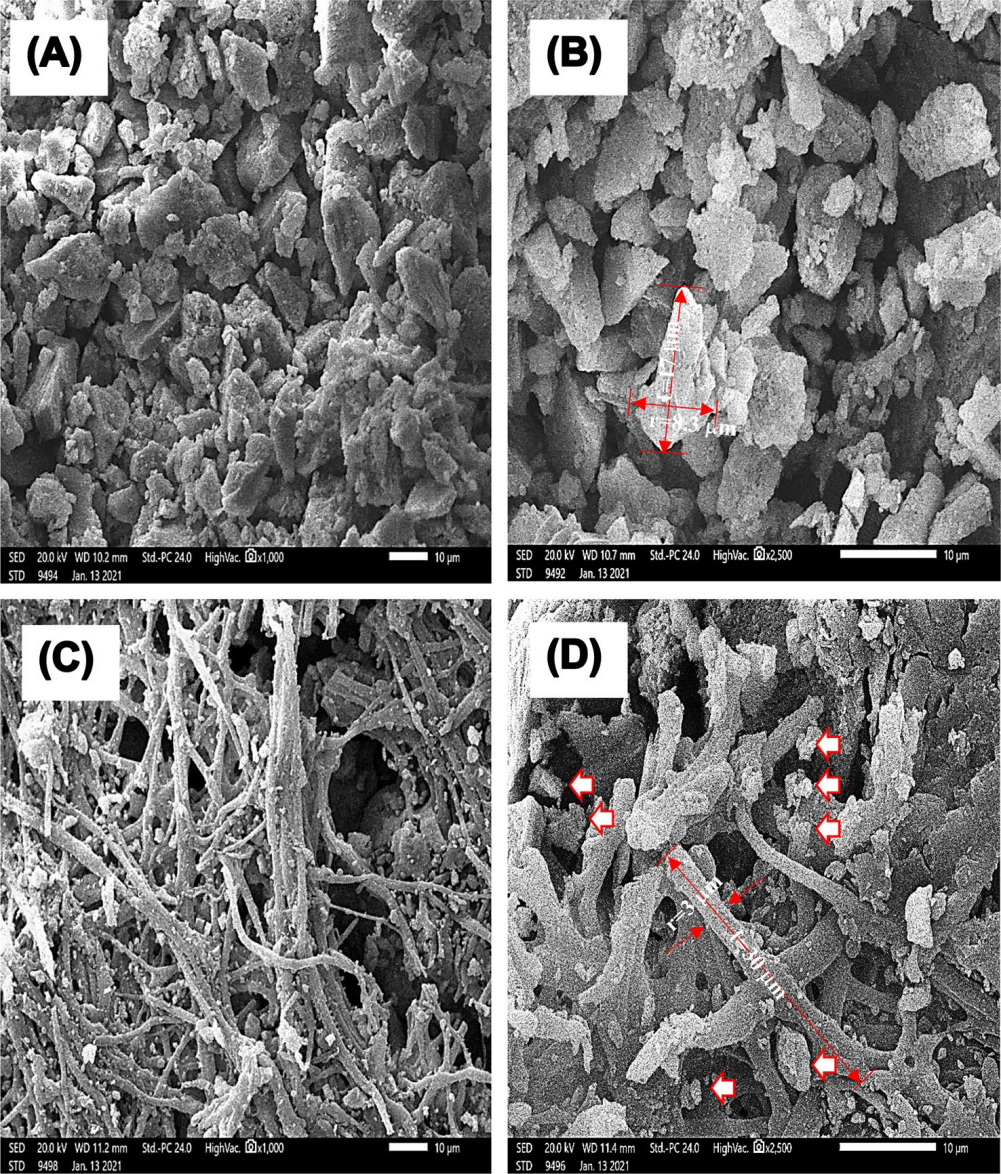
Scanning electron microscope (SEM) micrographs for the eggshell membrane (ESM) powder (**A**, **B**) and the ESM + Ulva *lactuca* mixture samples (**C**, **D**) at magnifications of 1,000*×* (**A** and **C**) and 2,500*×* (**B** and **D**)

SEM micrographs of Fig. 4A and B show the porous structure of the ESM powder samples, with an amorphous structure of irregular elongated particles (2–10 μm wide and 5–20 μm length), which was reported to be constituted of CaCO_3_ (94%), MgCO_3_ (1%), Ca_3_(PO_4_)_2_ (1%), and organic material (4%) [45]. The observed pores and cracks may occur and become more pronounced because of mechanical grinding, which also exposes CaCO_3_ in the form of crystal calcite [45, 46]. Micronized ESM biomaterials have shown a large surface area of 21.2 m^2^/g and porosity of 0.183 as compared to only 0.5 m^2^/g and 0.0009 for the raw eggshell [45], leading to enhanced skin contact, which promotes their efficacy as a wound healing treatment [15, 25, 46]. The ESM + Ulva *lactuca* mixture samples showed a highly homogenous porous structure of woven interconnected filaments (2–10 μm wide and 30–40 μm length) of Ulva *lactuca*, with ESM rough deposits in the pores among fibers (Fig. 4C and D). It is noteworthy that the rough texture of the mixture due to ESM particles may facilitate cell adhesion and proliferation [15]. In tissue engineering, adequate porosity in the targeted wound area is necessary for cellular uptake, migration, and survival; vascularization, gas exchange, nutrition delivery, and ingrowth into tissues [15, 47].

### Diabetic wound healing in vivo examination

**Fig. 5:**
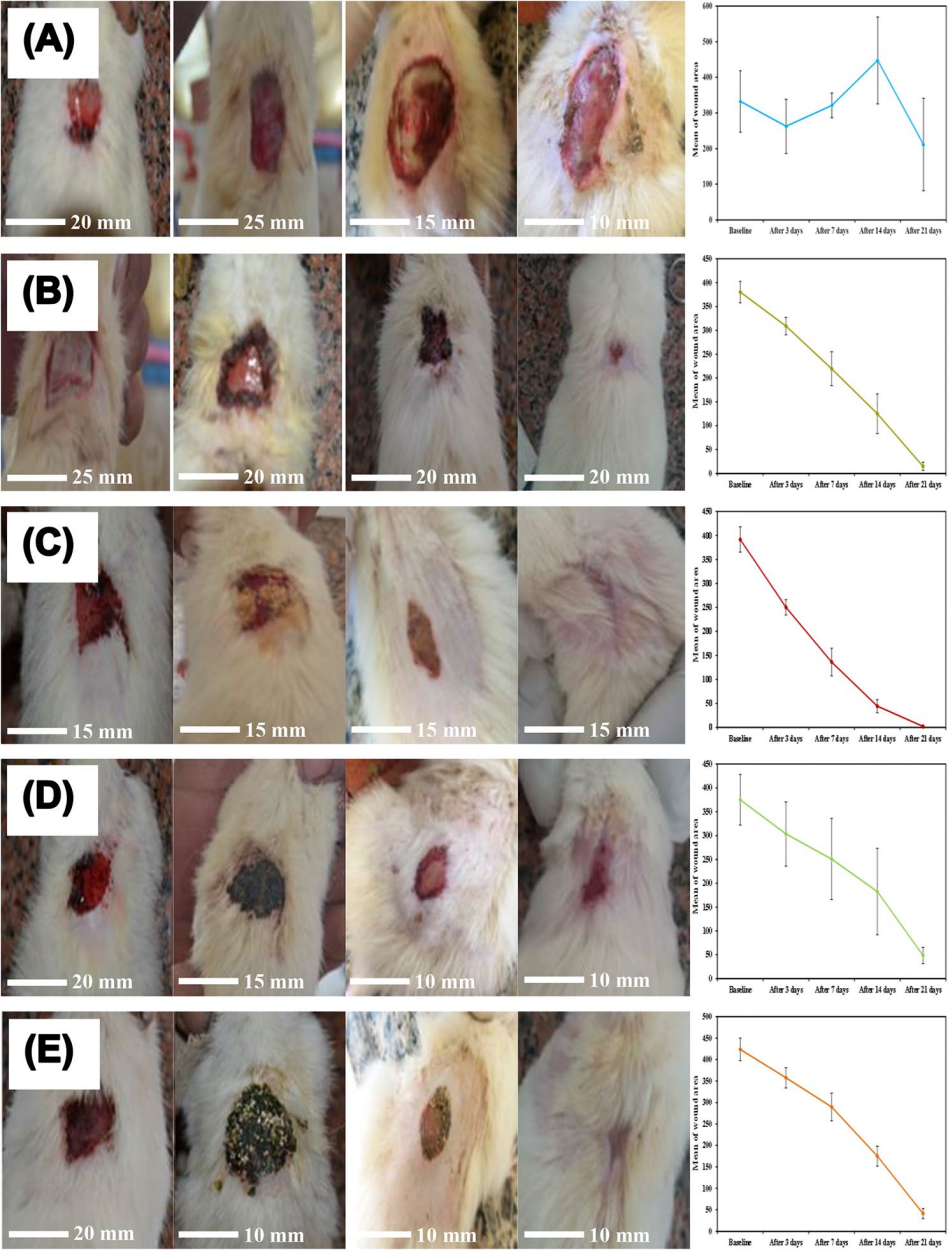
Stages of skin wound healing of diabetic untreated rats (negative controls, **A**) and treated with Dermazin cream (positive controls, **B**), eggshell membrane (ESM) powder (**C**), Ulva *lactuca* powder (**D**), and ESM + Ulva *lactuca* mixture (**E**), at baseline and after 3, 7, 14, and 21 days of treatment

Table 2; Fig. 5 show the size of skin wound area (mm^2^) for the diabetic untreated rats (negative controls) as compared to those treated with Dermazin cream (positive controls), ESM powder, Ulva *lactuca* powder, and ESM + Ulva *lactuca* mixture, at baseline and after 3, 7, 14, and 21 days of treatment. Wound sizes had significantly decreased in all rat groups that had wounds treated, but in the negative control group, it had grown larger by day 14 and day 21 (Fig. 5A). Figure 5B shows the wound healing stages for the positive control group treated with Dermazin, with a significant decrease in wound sizes from baseline to day 21. Dermazin, a 1% silver sulfadiazine cream, is a traditional antiseptic medication used to promote wound healing and maintain low bacterial colonization in burn wounds. It reduces bioburden, treats local infections, and prevents infection spreading throughout the body [48, 49].

**Table 2:** Comparison of wound area (mm^2^) for diabetic untreated rats (negative controls) and after treatment with Dermazin cream (positive controls), eggshell membrane (ESM) powder, Ulva *lactuca* powder, and ESM + Ulva *lactuca* mixture among the study groups, at baseline and after 3, 7, 14, and 21 days

	Negative Controls	Dermazin Cream	Eggshell Membrane	Ulva *lactuca*	Mixture
Baseline	332.6 ± 86.54	380.4 ± 22.30^*^	391.6 ± 26.32^*^	375.2 ± 53.08^*^	423.8 ± 26.31
Day 3	262.8 ± 75.84	309.0 ± 18.33^*^	250.4 ± 16.15	303.4 ± 67.47^*^	357.8 ± 23.75^*,a^
Day 7	321.6 ± 34.74	219.6 ± 35.65^*^	136.2 ± 28.73^*^	250.8 ± 85.25^*^	289.6 ± 32.36^*^
Day 14	447.6 ± 122.13^a^	125.2 ± 41.52^*^	44.20 ± 13.68^*^	182.6 ± 90.74^*^	175.0 ± 23.43^*^
Day 21	211.4 ± 130.0^a^	14.80 ± 8.53^*,b^	2.00 ± 1.22^*,b^	48.60 ± 17.02^*,b^	41.40 ± 11.99^*,b^

*P < 0.001 as compared to the negative control group

Wound closure quantitative analysis showed that the ESM group healed at a rate of 99.49%, with diabetic wounds completely closed and hair growing again on day 21, which was significantly (*p* < 0.001) faster than the other groups (Fig. 5C). The negative control group reached a final healing rate of 36.44%, the positive control group 96.79%, the Ulva *lactuca* group 87.05%, and the mixture group 90.23% (Table 2; Fig. 5). Thus, the ESM treatment outperformed Dermazin, Ulva *lactuca*, and the mixture treatments by a significant margin (*p* < 0.001). The ESM treatment results also outperformed those by Choi et al. [50], who showed that raw ESM marginally enhanced skin wound healing compared to negative control rats and that acid-modified ESM could improve tissue regeneration by promoting a full-thickness epidermal layer for faster wound healing. In an excisional wound splinting hairless mice model, soluble ESM accelerated wound closure at days 3, 7, and 10 through fibroblast-to-myofibroblast differentiation, enhancing fibroblast proliferation and the alpha-smooth muscle actin (α-SMA) contractile protein levels in vitro [25]. Granulation tissue myofibroblasts have been shown to promote wound closure by bringing the wound edges closer together through exerting tension and contracting the extracellular matrix [13].

### Histological analysis

**Fig. 6:**
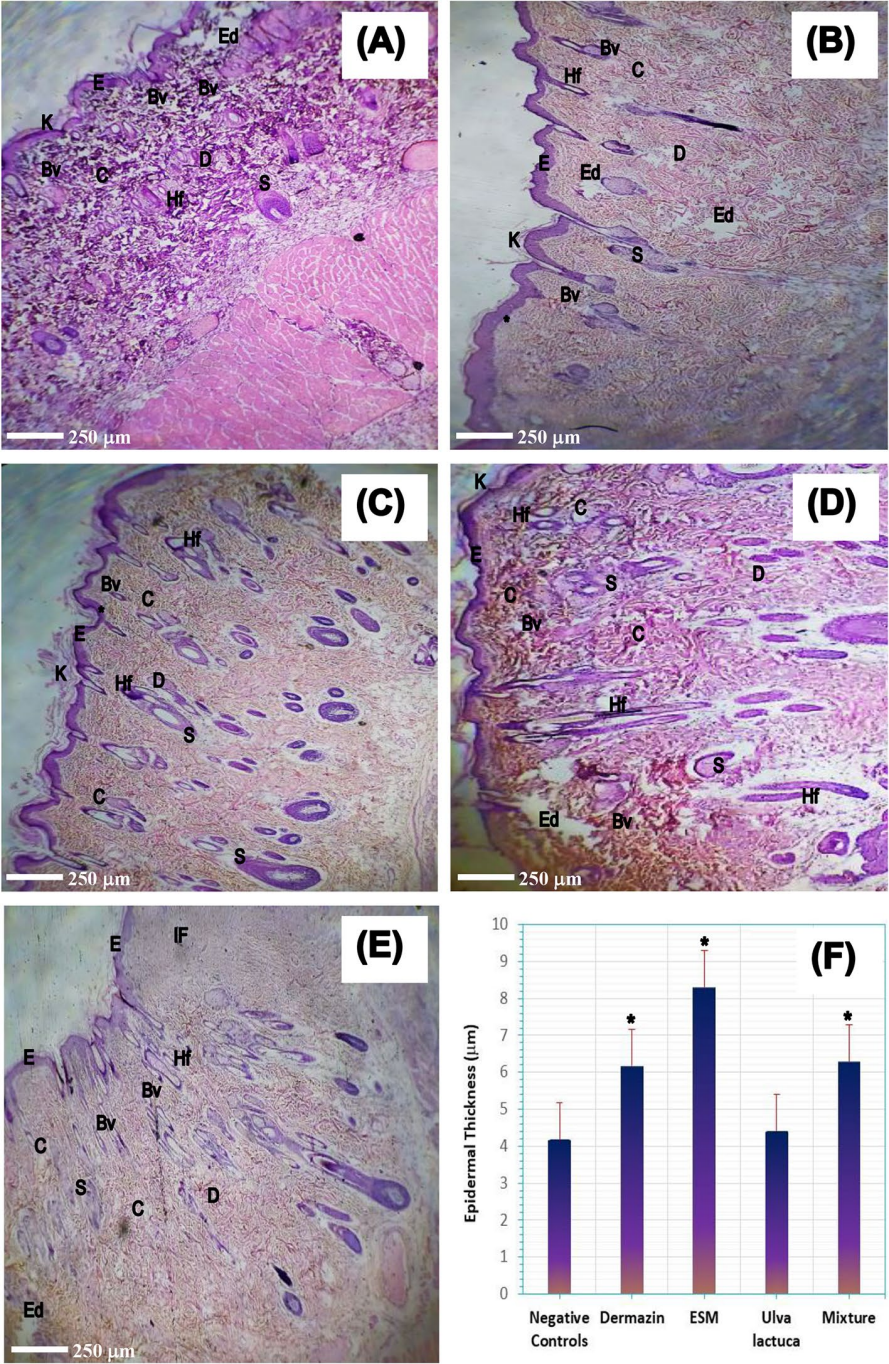
Histological evaluation of skin wound healing of diabetic untreated rats (negative controls, **A**) and treated with Dermazin cream (positive controls, **B**), eggshell membrane (ESM) powder (**C**), Ulva *lactuca* powder (**D**), ESM + Ulva *lactuca* mixture (**E**), and the epidermal thickness (in µm) of each group is shown as bar chart (*p < 0.001 as compared to negative controls, **F**). [H&E staining original magnification 200*×*, Keratinocytes (K), Epidermal layer (E), Dermis layer (D), Blood vessel (Bv), Collagen fibers (C), Hair follicles (Hf), Sebaceous gland (S), Edema (Ed), Infiltrating lymphocytes (IF), and Basement membrane (*)]

Successful skin wound healing within a reasonable time frame entails four consecutive and overlapping phases: hemostasis, inflammation, proliferation, and remodeling. During the proliferative phase, the skin undergoes angiogenesis, collagen deposition, tissue granulation, and re-epithelization formation [9, 10, 14, 15, 25, 35–37]. The histological examination at a magnification of 200× and the qualitative histopathological analysis of the skin wound healing of diabetic rats after applying Dermazin cream (positive control), ESM powder, Ulva *lactuca* powder, and ESM + Ulva *lactuca* mixture as compared to the untreated negative control rats are shown in Fig. 6; Table 3.

**Table 3:** Qualitative histological analysis of skin wound healing for diabetic untreated rats (negative controls) and after treatment with Dermazin cream (positive controls), eggshell membrane (ESM), Ulva *lactuca*, and ESM + Ulva *lactuca* mixture powders of all study groups at day 21

	Negative Controls	Dermazin Cream	Eggshell Membrane	Ulva *lactuca*	Mixture
Re-epithelization	++	+++	++++	++	++
Pigments	+	+++	+++	+	++
Keratinocytes	+	++	+++	+	++
Well basement membrane	++	+++	++++	+	+++
Infiltrating lymphocytes	+++	++	+	++	++
Organized collagen fibrines	+	++	++++	+	++
Congested blood vessels	++	+	+	++	++

The negative control rats showed weak wound healing, with irregular re-epithelialization and disassociated keratinocytes lining the upper surface of the epidermal layer (Fig. 6A). The dermal layer had an abundance of blood capillaries, dilated blood vessels, collagen fibrin, and a sparse distribution of edemas. The wounds also had a broad region of edema and pale dark pigments on the epidermis. The positive control group of rats treated with Dermazin cream had significantly better wound healing, with less epithelial necrotic cell infiltration and better re-epithelialization of the epidermal layer (Fig. 6B). The basement membrane was enfolded, and lymphocytes were minimal, with sebaceous gland and hair follicles migrated to the upper epidermal layer. The dermis layer had irregular perpendicular collagen fibrins, and dilated blood vessels and blood capillaries leading to edema.

Wound healing in rats treated with ESM was significantly better than in control rats. This improvement was accompanied by a well-formed basement membrane, well differentiated epithelial cells, and regular thick keratinocytes lining the surface of the epidermal cells (Fig. 6C). The wounds showed moderately dilated blood vessels and blood capillaries perpendicular to the epidermal layer, which was covered with regular collagen fibrins in different directions, and the sebaceous gland migrated to the upper epidermis. These results are consistent with earlier findings showing that soluble ESM topical treatment could enhance skin wound healing in an excisional wound splinting mouse model, by stimulating the synthesis of collagen type III in the papillary dermis of hairless mice [13, 25, 51]. During the early stages of healing, resident and myeloid cell-converted fibroblasts primarily synthesize collagen type III, creating a flexible matrix that supported cell migration and granulation tissue formation. However, it is eventually replaced by collagen type I in healed skin [52]. Lysyl oxidase enzyme-induced covalent cross-linking during granulation tissue formation could promote remodeling and collagen fibril assembly, restoring tensile strength to over 80% of normal tissue for months following wound closure [25, 37, 52, 53]. The elasticity and reversible deformation characteristics of these collagen fibrils were affected by cross-links mediated by disulfide bonds, reducible and mature cross-links, transglutaminase cross-links, and advanced glycation end products [52]. An in vitro investigation found that human dermal fibroblasts cultured with soluble ESM triggered genes encoding collagen type III, which improved the papillary dermis health, increasing skin elasticity and alleviating the wrinkles in humans [54].

Although, ESM per se regulated inflammation and accelerated cell proliferation, angiogenesis, and wound contraction [16], bioactive glass nano-coatings with copper could modify the ESM’s dielectric and physicochemical properties leading to the formation of a continuous and homogeneous layer of the epidermis in vivo [13]. EMS and silver nanoparticle composites also promoted cell proliferation while suppressing inflammation, leading to improved re-epithelialization, granulation tissue formation, and wound healing in Albino mice [12, 13, 17, 55]. ESM powders have been found to stimulate matrix metalloproteinase (MMP) activity in mice dermal fibroblasts, increase MMP-2 protein levels, and accelerate the α-SMA contractile protein synthesis, thereby promoting wound healing and keratinocyte cell proliferation [13, 25, 51]. MMPs are important regulators for angiogenesis, tissue homeostasis, and immune response by modulating the release or activation of chemokines, cytokines, and growth factors [51, 52]. MMP activity, which regulates cell surface recruitment, substrate availability, and protein interactions, is tightly regulated at multiple levels, including transcriptional control, mRNA stability, pro-peptide activation, and proteolysis inhibition by tissue inhibitors [56]. It has been shown that biofilm-mediated microRNAs upregulate MMP-2, leading to a collagenolytic environment in wounds, resulting in a decrease in collagen I/collagen III ratio, which affects the biomechanical properties of repaired skin [52].

ESM is a naturally occurring biopolymer with 90% structural proteins, 3% lipids, and 2% carbohydrates. Proteome analysis, based on total spectral counts of unique peptides, showed that processed ESM powders are primarily composed of cysteine-rich proteins (∼ 30%) and collagen type X (10%), along with the bioactive enzymes lysyl oxidase-like protein 2 (30%) and lysozyme C (10%), involved in collagen processing [25, 53]. These constituents had favored ESM powders to regulate MMPs on fibroblasts in vitro and on epidermal cells in vivo, promoting cell proliferation and myofibroblast differentiation due to re-epithelialization and fibroblasts activation [34, 51]. ESM promoted fibroblast proliferation after 3 days of incubation without affecting cell viability, suggested biocompatibility, and continued stimulation upon further incubation [51]. Fibroblasts, which are normally dormant, play a vital role in wound healing upon activation by producing matrix components essential for wound contraction and closure. After tissue injury, activated fibroblasts proliferate in the peri-wound stroma and migrate into the wound provisional matrix of fibrin and fibronectin [34]. This proliferation, expansion, migration, and myofibroblast differentiation are essential for granulation tissue formation and healing, requiring membrane-bound molecules like integrin and syndecan-4 [35].

On the other hand, the Ulva *lactuca* treated rats showed weaker wound healing, with few keratinocytes covering the upper surface of a thin epidermal layer (marked by dark pigments), necrotic epithelial and lymphocytic cells infiltrating the basement membrane (Fig. 6D). Multiple necrotic sebaceous glands encircled the hair follicles in the dermis layer, which also had dilated blood vessels, congested capillaries, and migrated clotting collagen fibrins in different directions. In contrast, rats treated with ESM + Ulva *lactuca* mixture showed mild healing comparable to ESM and Dermazin treatments (Fig. 6E). Secondary metabolites found in Ulva *lactuca* include steroids, alkaloids, triterpenoids, saponins, phenolic compounds, and flavonoids, which are anti-inflammatory, antioxidant, anticoagulant, and antimicrobial activities and have proven crucial for wound healing [20–22, 57]. However, compared to Dermazin or ESM treatments, the rates of re-epithelialization, collagen formation, tissue construction, and wound healing were much lower when using either Ulva *lactuca* extracts alone or mixed with EMS powder (Table 3). We hypothesize that this is because Ulva *lactuca* increases the mixture’s dielectric constant to significantly higher levels (Fig. 3), which in turn alters its net negative charges, conduction ionic minerals, and intrinsic phytochemicals necessary for tissue regeneration. Flavonoids are also known to enhance collagen fibril viability, prevent cell damage, stimulate DNA synthesis, and promote fibroblast cell proliferation and differentiation into specialized myofibroblasts, leading to wound contraction and closure [58]. Triterpenes, which are absent in Ulva *lactuca*, have been shown to induce cell migration, proliferation, and collagen deposition by regulating reactive oxygen species generation, thus accelerating the wound healing process [59, 60]. Moreover, Ulva *lactuca* has a higher lipid and a lower protein concentration compared to ESM, which exhibited weak antiproliferative capacities [61]. These reasons may explain why rats treated with Ulva *lactuca* alone or with ESM + Ulva *lactuca* mixture had a slower healing rate and wound closure compared to those treated with Dermazin or ESM.

Finally, ESM treatment resulted in a significantly thicker epidermal layer as compared to Dermazin, ESM + Ulva *lactuca* mixture, and Ulva *lactuca* treatments and to negative controls (i.e., 8.30 ± 0.70 vs. 6.17 ± 1.44, 6.29 ± 0.68, 4.41 ± 0.77, and 4.17 ± 0.73 μm, *p* < 0.001, respectively) (Fig. 6F). In a recent study conducted on male Albino rats, the use of ESM powders and dressing significantly improved tissue regeneration after superficial open wounds. Compared to conventional treatment using hydrogen peroxide, which resulted in a length reduction rate of 0.852 mm/day and healing in 16 days, the ESM and dressing treatments resulted in a length reduction rate of 1,009 and 1,020 mm/day, respectively, and shortened the healing time to 12 days [14]. Thus, ESM powder is a powerful biomaterial for tissue engineering and chronic wounds healing, such as diabetic venous, foot, and pressure ulcers [3, 4, 16].

In conclusion, nonhealing diabetic wounds cause global morbidity and poor quality of life. Understanding wound healing mechanisms is crucial for developing effective treatments. ESM, a natural waste product with proteinaceous meshwork, is a potential biomaterial for treating chronic wounds. In the present study, ESM, Ulva *lactuca*, and their mixture micronized powders were evaluated for compressive strengths, dielectric properties, and SEM surface morphology, which showed stable favorable characteristics that could promote in vivo wound healing in a rat model of induced diabetes. Wound closure was significantly hastened on days 3, 7, and 14 with ESM treatment, and epidermal layer was much thicker with enhanced re-epithelization and collagen deposition on day 21, compared to conventional Dermazin and Ulva *lactuca* treatments. It is therefore postulated that ESM’s structural extracellular matrix-like components promoted re-epithelialization and neovasculature development, leading to better wound healing. Thus, ESM is a practical biomaterial for the design of innovative wound healing remedies that are both economical and effective.

## Data Availability

Metadata used and/or analyzed during the current study will be made available from the corresponding author on reasonable request.

## Abbreviations

α-SMA: Alpha-Smooth muscle actin
ESM: Eggshell membrane
H&E: Hematoxylin and eosin
LCR: Inductance, capacitance, and resistance
MMP: Matrix metalloproteinase
